# Predictors of growth patterns in children with mucopolysaccharidosis I after haematopoietic stem cell transplantation

**DOI:** 10.1002/jmd2.12291

**Published:** 2022-04-26

**Authors:** Stefanie Maier, Miroslav Zivicnjak, Lorenz Grigull, Julia B. Hennermann, Charlotte Aries, Britta Maecker‐Kolhoff, Martin Sauer, Anibh M. Das, Rita Beier

**Affiliations:** ^1^ Department of Paediatric Haematology and Oncology Hannover Medical School Hannover Germany; ^2^ Department of Paediatric Kidney Liver and Metabolic Diseases at Hannover Medical School Hannover Germany; ^3^ Rare Disease Centre, Bonn University Medical Centre Bonn Germany; ^4^ Villa Metabolica, Department of Paediatric and Adolescent Medicine University Medical Centre Mainz Germany; ^5^ Department of Paediatrics Hamburg‐Eppendorf University Medical Centre Hamburg Germany

**Keywords:** anthropometry, children, growth pattern, haematopoietic stem cell transplantation, mucopolysaccharidosis I

## Abstract

Mucopolysaccharidosis type I (MPS I) is an autosomal‐recessive metabolic disorder caused by an enzyme deficiency of lysosomal alpha‐l‐iduronidase (IDUA). Haematopoietic stem cell transplantation (HSCT) is the therapeutic option of choice in MPS I patients younger than 2.5 years, which has a positive impact on neurocognitive development. However, impaired growth remains a problem. In this monocentric study, 14 patients with MPS I (mean age 1.72 years, range 0.81–3.08) were monitored according to a standardised follow‐up program after successful allogeneic HSCT. A detailed anthropometric program was carried out to identify growth patterns and to determine predictors of growth in these children. All patients are alive and in outpatient care (mean follow‐up 8.1 years, range 0.1–16.0). Progressively lower standard deviation scores (SDS) were observed for body length (mean SDS −1.61; −4.58 – 3.29), weight (−0.56; −3.19 – 2.95), sitting height (−3.28; −7.37 – 0.26), leg length (−1.64; −3.88 – 1.49) and head circumference (0.91; −2.52 – 6.09). Already at the age of 24 months, significant disproportions were detected being associated with increasing deterioration in growth for age. Younger age at HSCT, lower counts for haemoglobin and platelets, lower potassium, higher donor‐derived chimerism, higher counts for leukocytes and recruitment of a matched unrelated donor (MUD) positively correlated with body length (*p* ≤ 0.05). In conclusion, this study characterised predictors and aspects of growth patterns in children with MPS I after HSCT, underlining that early HSCT of MUD is essential for slowing body disproportion.

**Synopsis**

We identified new predictors for subsequent growth in children with mucopolysaccharidosis type I (MPS I) after haematopoietic stem cell transplantation (HSCT).

## 1 INTRODUCTION

Type I mucopolysaccharidosis (MPS I) is an autosomal‐recessive metabolic disorder caused by an enzyme deficiency in lysosomal alpha‐l‐iduronidase (IDUA), which leads to accumulation of glycosaminoglycans (GAGs). Based on severity of symptoms, three clinical phenotypes are distinguished: severe Hurler, intermediate Hurler–Scheie and attenuated Scheie syndrome. The clinical manifestation includes hydrocephalus, cardiac disease, airway compromise, hepatosplenomegaly, psychomotor delay and hearing loss.^1^, ^2^ Musculoskeletal involvement is characterised by abnormal growth, dysostosis multiplex, severe skeletal deformities and degenerative joint disease.^3^

Therapeutic options to modify disease in MPS I include haematopoietic stem cell transplantation (HSCT) and enzyme replacement therapy (ERT) with recombinant alpha‐iduronidase. As ERT does not cross the blood–brain barrier, it does not sufficiently stabilise cognitive or central nervous system function.^4^ Early diagnosis and HSCT at young age are the appropriate treatment.^5^, ^6^ It is the only therapy that allows long‐term survival and prevents or delays complications including neuropsychological impairment.^7^, ^8^ Skeletal deformities, however, are not amenable to HSCT and continue to progress.^9^ MPS I leads to impaired growth and development.^10^ The disease may cause a sequence of pathological processes, which promote inflammation, cartilage degradation and hyperplasia of synovial membranes resulting in poorly organised and metabolically abnormal connective tissue matrices.^11^ In future, for children with MPS I gene therapy might be an option.^12^

Monitoring growth parameters of these children is critical in assessment of disease progression and therapeutic success. Nevertheless, the body of data on factors that impact growth patterns after HSCT in these children are limited. Only head circumference and body length have traditionally been measured.^13^ The objective of this study was to execute a detailed program capturing growth patterns of patients with MPS I after HSCT and define predictors of growth.

## 2 METHODS

### 2.1 Patients

This was a mono‐centric, prospective, longitudinal, non‐randomised study. Fourteen patients (eight female, six male) with MPS I underwent HSCT at Hannover Medical School between 2001 and 2018. Mean age at HSCT was 1.72 years (range 0.81–3.08). Diagnosis was confirmed by clinical picture and enzyme deficiency and/or genetic analysis. If age at diagnosis was >2 years, paediatric neurologists and HSCT team discussed proceeding to HSCT with the parents after neurocognitive evaluation. Characteristics of included patients, detailed information about HSCT and a description of the orthopaedic pathologies are summarised in additional material (Tables S1–S3). Patient 13 received a second HSCT because of graft failure after HSCT; there we used second HSCT for further analysis. Patients were transplanted using the MPS‐HCT 2005 preparative regime.^14^, ^15^

### 2.2 Data collection

In addition to standard HSCT follow‐up procedures, a detailed anthropometric program was implemented, which was in accordance with standardised technique as recommended by the ‘International Biological Program’.^16^ Body length, sitting height, leg length, head circumference and body weight were recorded and compared to age‐ and gender‐specific reference data as standard deviation scores (SDS), which had been measured for 5155 healthy children aged 3–18 years.^17^ SDS for birth to 2 years were calculated from KiGGS 2003–2008.^18^

### 2.3 Ethical considerations

The study was approved by the Ethics Committee of Hannover Medical School (#3701). Informed consent was obtained from patients and/ or their legal representative as appropriate.

### 2.4 Statistical analysis

SDS values for the examined parameters were calculated according to the equation SDS = (*x*_i_ – *x*_s_) /SD. Where *x*_i_ represents the individual value of patient, *x*_s_ and SD are the mean and SDS of the reference group, respectively. The normality of distribution was assessed for each age cohort and each observed parameter (Kolmogorov–Smirnov test). Data are presented as mean and 95% confidence interval. All measurements were grouped according to age at examination. The linear‐mixed effects models were used for the assessment of age and donor‐related changes in linear growth of MPS I children after HSCT (mixed procedure in SPSS software, version 26). Statistical significance was assumed at *p* ≤ 0.05.

## 3 RESULTS

### 3.1 Patients

All patients who received HSCT for MPS I are alive and in outpatient care. The mean follow‐up time was 8.1 years (range 0.1–16.05). Ten patients received HSCT from matched unrelated donor (MUD), while two patients were transplanted from matched sibling donors (patients 8 and 12) and two patients from haplo‐identical donors (patients 4 and 7). Eight patients presented with full donor chimerism (donor cells ≥94%), while six patients exhibited intermediate mixed chimerism (last chimerism in this cohort 85%–100%). Engraftment and GvHD data are shown (Table S4). Renal, thyroid function, growth hormone (GH) parameters, sexual hormones (measured in 7/14 patients), age at menarche and enzyme activity were normal in all patients (Table S5). No patient has received treatment for hypo‐ or hyperthyroidism, nor GH replacement.

### 3.2 Infancy

**FIGURE 1:**
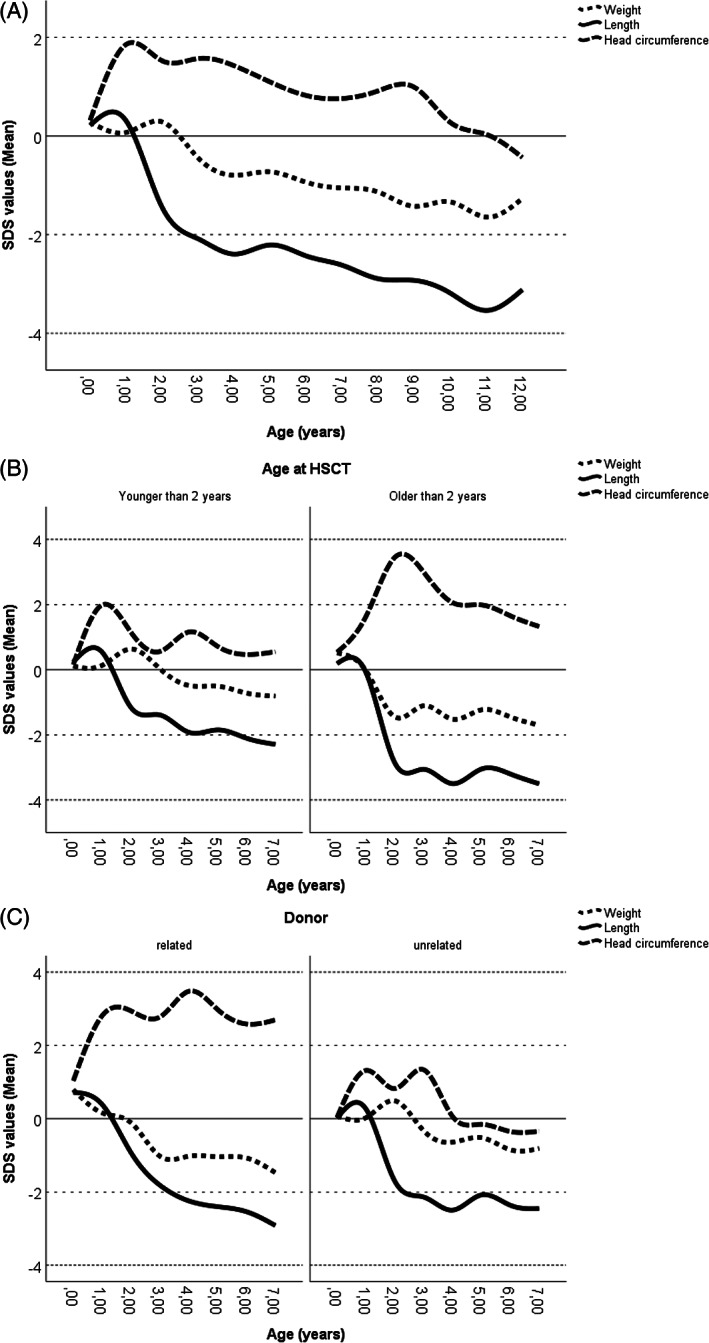
Distribution of mean SDS relative to controls for weight, body length and head circumference in (A) the whole cohort, (B) cohort split by age at HSCT and (C) cohort split by donor type

At birth, body length, weight and head circumference were similar to the reference group. There is a further increase in head circumference SDS from 6 to 24 months, while body length and weight did not rise in this period. Relating growth development to age at HSCT, there is an increasing trend for disproportion in as yet untreated children at the age of 24 months, with increased weight and decreased length. At the same age, all parameters presented (Figure 1) recovered in transplanted patients.

Regarding the impact of donor type on growth development, growth was less disproportionate in patients receiving HSCT from a MUD than in those who were transplanted from a related donor (RD): this is particularly evident in greater SDS for head circumference SDS (Figure S1, Table S6).

### 3.3 Age‐related body proportions and growth

**FIGURE 2:**
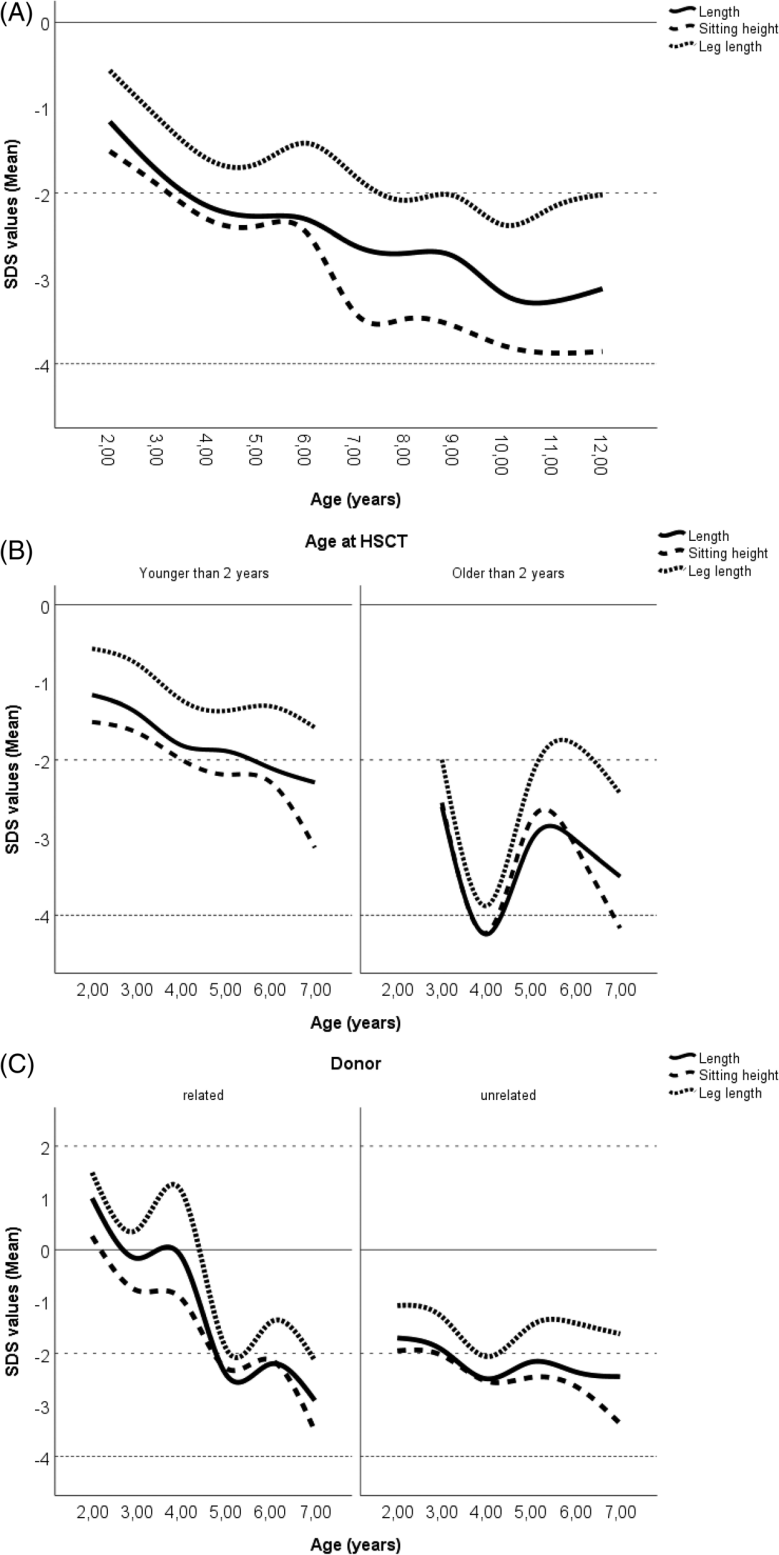
Distribution of mean SDS relative to control for body length, sitting height and leg length in (A) whole cohort, (B) cohort split by age at HSCT and (C) cohort split by donor type

Figure 1 shows the mean SDS for weight, body length and head circumference from birth to the age of 12 years. Figure 2 presents age‐related body dimensions (body length, sitting height and leg length) from 2 to 12 years of age (estimated marginal means in Table S7). In general, growth parameters in MPS patients decline over time and deterioration in linear growth manifests primarily in increasingly poor sitting height (mean SDS weight − 0.56 (range − 3.19 – 2.95), mean body length SDS − 1.61 (range − 4.58 – 3.29), mean SDS sitting height − 3.28 (range − 7.37 – 0.26), mean SDS leg length − 1.64 (range − 3.88 – 1.49)). Greater SDS for head circumference in infancy (mean SDS 0.91 [range − 2.52 – 6.09]) approached the reference group with increasing age.

Figures 1B and 2B show effects of age at HSCT: SDS values were worse, when patients were older than 2 years at HSCT. Age at HSCT has statistically significant effect on weight (*p* < 0.04), body length (*p* < 0.01) and sitting height (*p* < 0.03).

Figures 1C and 2C differentiate between donor types. Growth parameters fell with age in both groups to similar extent. There was a clear decrease in all parameters at the age of 4 years in children receiving HSCT from RD. Greater SDS for head circumference appeared especially for patients receiving HSCT of RD.

### 3.4 Predictors of linear body dimensions

There was a significant positive correlation of younger age at HSCT, lower haemoglobin, lower platelet count, lower potassium, higher chimerism, higher leukocyte count and donor type with length SDS (*p* < 0.05, respectively), while enzyme activity of IDUA was not a significant predictor (Table 1). The sitting height index is the ratio between trunk length and total body height. A correlation between measured haemoglobin and current sitting height index (z‐score) is attached (Figure S2). A tendency to normalisation of sitting height index was found when haemoglobin concentrations were lower.

**TABLE 1:** Linear mixed‐effects models of predictors of length SD scores in 14 patients after HSCT

Covariate	Estimate	SE	95% Confidence interval	*p*‐value
Age at transplantation	−3.58	0.74	−5.18 to −1.99	<0.01
Chimerism^a^	0.10	0.04	0.02 to 0.19	0.03
Haemoglobin^a^	−0.99	0.23	−1.49 to −0.48	<0.01
Leukocytes^a^	0.48	0.13	0.21 to 0.75	<0.01
Platelets^a^	−0.01	0.00	−0.01 to −0.002	0.01
Potassium^a^	−1.37	0.33	−0.64 to −2.10	<0.01
Enzyme activity^a^	0.06	0.10	−0.15 to 0.27	0.50
Type of donor	1.38	0.39	0.55 to 2.21	<0.01

*Note*: Data are presented as estimated marginal means (95% confidence intervals); p values are based on the linearly independent pairwise comparisons among the estimated marginal means.

^a^ Measured at all follow‐up, linear mixed‐effects models include all measured values.

## 4 DISCUSSION

This is the first study using standardised anthropometric procedures to measure a wide spectrum of growth parameters in MPS I patients after HSCT. Before and soon after HSCT, growth pattern for MPS I showed the following: (1) children had a normal growth pattern at birth, (2) in comparison to the reference population, abnormal growth patterns were observed 4–6 months after birth (before HSCT), resulting in the significant disproportion of body dimensions at the end of infancy, (3) head circumference was above normal, especially for patients receiving HSCT of RD.

Some of these aspects were well known in children with MPS I after HSCT: Body length at birth was similar or increased compared to reference groups.^19^ One possible explanation for normal or increased growth in early life might be that heparan sulphate binds as co‐receptor to proteins— including growth factors. Increased levels of heparan sulphate might overstimulate axial bone growth.^20^ An enlarged head circumference is a well‐known feature of MPS I patients.^21^ Thickened calvaria, accumulation of GAGs and sphingolipids in neurons and neuroinflammation may be responsible for the phenotype.^22^ Kiely reported MPS I patients with an enlarged head circumference at median age of 8 months.^23^ Because of its early manifestation, higher‐to‐normal head circumference is often the key feature leading to diagnosis.^13^ This could be observed even slightly earlier in our cohort and underlines that the alteration in growth patterns begins considerably earlier than previously assumed.

Long‐term follow‐up in our study population gave new insights: (1) after initial stabilisation, longitudinal growth slowed down, (2) poor longitudinal body growth was associated with significant disproportion between sitting height and leg length, which resulted in disproportionate growth pattern for body length and (3) SDS values for body length were more favourable when MUD were available for HSCT, patients were younger at HSCT, achieved higher donor‐derived haematopoietic chimerism, better leucocytes counts, lower counts for haemoglobin, platelets and lower serum potassium levels.

It was reported that median length falls below the third percentile in untreated MPS I patients.^21^ Although HSCT had positive impact on several key features and an initial period of catch‐up growth is observed, it is followed by constant slowing down of growth in later childhood.^24^ There are variable signs of dysostosis multiplex and skeletal dysplasia despite of HSCT,^9^, ^25^ which might have important effects on restricted linear growth. Genua valga, hip and spinal deformities may contribute to short leg length and sitting height and therefore lead to major reductions in body length. Sitting height and leg length were not separately addressed in previous studies.^13^, ^19^, ^21^ We found that the change in sitting height had the most prominent impact on compromised linear growth. This non‐invasive parameter may be influenced by scoliosis and has less side effects than procedures employing X‐ray. In transplanted patients before age of 2 years, we observed more normal SDS of all measured parameters, less disproportion and more harmonisation. Thus, early HSCT is not only essential to provide a possibility for improved neurologic development but also might have beneficial effect concerning the musculoskeletal involvement.

In our study, HSCT of MUD had positive impact on different length parameters, which might be due to higher secretion of IDUA. As in our study, we are reporting about long time frame of HSCT (2001–2018), donors were not pre‐screened for enzyme activity, so there might even be heterozygous donors in the RD cohort, which is associated with poorer outcome in body length as already published.^6^

We confirmed that chimerism and age at HSCT influenced growth, but failed to find an effect of the underlying genotype or post‐HSCT enzyme activity. Other studies reported an association of genotype and post‐treatment IDUA with dysostosis multiplex,^26^ which contributed to shorter body length. This could be caused by GH deficiency, which is often diagnosed after HSCT.^27^ A treatment with GH might be discussed, but due to its long‐term outcome is not fully described yet,^28^, ^29^ it was not in our cohort. Long‐term consequences of transplant conditioning to growth were also described.^30^, ^31^ The latter factors may be related to degree of chimerism and donor type, which is in line with our data.^32^ Our small cohort might explain absence of a significant effect of enzyme activity on body length. As leucocytes of transplanted patients are the only site of production of IDUA, they may be significantly associated with length. Higher SDS values for length were associated with higher leucocyte levels. In addition to chimerism and leucocyte count, several predictors of linear body growth after HSCT were identified in this study: age at HSCT, haemoglobin, potassium, platelets and donor type.

Lower haemoglobin was associated with higher length SDS. A possible explanation for this phenomenon may be compromised respiratory function in severely affected patients with scoliosis which results in poor oxygenation and increased haemoglobin concentrations.^33^ Because of the deterioration in ventilation, respiratory acidosis may develop, with metabolic compensation.^34^ Potassium is an indirect parameter of renal regulation, and tends to compensate for respiratory acidosis. Therefore, lower potassium may be associated with higher length SDS due to the putative lower respiratory acidosis.

Lower platelet count correlated with higher length SDS. Arterial hypertension and stenosis are known in MPS I patients. Histopathological examination presented proliferation of collagen and elastic fibres and also not degradable mucopolysaccharides with markedly distended lysosomes.^35^ Thus, we suspect lower platelet count to prevent cardiovascular events. These aspects (haemoglobin, potassium and platelets) should be examined in greater cohorts, to get more insight in the potential pathophysiology.

This study is limited by its small cohort with MPS I. Pulmonary function tests have not been conducted in all children due to poor compliance in small children. Pubertal status and bone age were not sufficiently evaluated. Unfortunately, we did not record the course of enzyme activity levels for all children after HSCT, which would be interesting even over time.

## 5 CONCLUSION

This study characterised new predictors and aspects of growth patterns in children with MPS I after HSCT and underline the need for dedicated long‐term follow‐up. These patients presented a progressive imbalance in body proportions. Younger age at HSCT, lower haemoglobin, lower platelet count, lower potassium, higher chimerism, higher leukocyte count and donor type were significantly correlated with body length SDS.

Future studies are warranted to study these effects in greater cohorts and to provide more detailed analysis. This should increase our understanding of disease progression and therapeutic success in MPS I children.

## CONFLICT OF INTEREST

All authors do not declare financial disclosures appropriate to have influence on the direction of the paper. RB received financial support for an advisory board by BluebirdBio regarding thalassemia patients, and congress travel costs by MEDAC and NEOVII

## Supporting information


**Data S1** Supporting information

## References

[1] Clarke LA , Giugliani R , Guffon N , et al. Genotype‐phenotype relationships in mucopolysaccharidosis type I (MPS I): insights from the international MPS I registry. Clin Genet. 2019;96:281‐289. 31194252 10.1111/cge.13583 PMC6852151

[2] Beck M . Variable clinical presentation in lysosomal storage disorders. J Inherit Metab Dis. 2001;24:47‐51. 11758678 10.1023/a:1012463605992

[3] White KK . Orthopaedic aspects of mucopolysaccharidoses. Rheumatology. 2011;50(5):26‐33. 10.1093/rheumatology/ker393 22210667

[4] Shapiro EG , Eisengart JB . The natural history of neurocognition in MPS disorders: a review. Mol Genet Metab. 2021;133:8‐34. 33741271 10.1016/j.ymgme.2021.03.002

[5] De Ru MH , Boelens JJ , Das AM , et al. Enzyme replacement therapy and/or hematopoietic stem cell transplantation at diagnosis in patients with mucopolysaccharidosis type I: results of a European consensus procedure. Orphanet J Rare Dis. 2011;6:55. 21831279 10.1186/1750-1172-6-55 PMC3170181

[6] Aldenhoven M , Wynn RF , Orchard PJ , et al. Long‐term outcome of hurler syndrome patients after hematopoietic cell transplantation: an international multicenter study. Blood. 2015;125:2164‐2172. 25624320 10.1182/blood-2014-11-608075

[7] Aldenhoven M , Jones SA , Bonney D , et al. Hematopoietic cell transplantation for Mucopolysaccharidosis patients is safe and effective: results after implementation of international guidelines. Biol Blood Marrow Transplant. 2015;21(6):1106‐1109. 25708213 10.1016/j.bbmt.2015.02.011

[8] Taylor M , Khan S , Stapleton M , et al. Hematopoietic stem cell transplantation for Mucopolysaccharidoses: past, present, and future. Biol Blood Marrow Transplant. 2019;25(7):e226‐e246. 30772512 10.1016/j.bbmt.2019.02.012 PMC6615945

[9] Field RE , Buchanan JAF , Copplemans MGJ , Aichroth PM . Bone‐marrow transplantation in Hurler's syndrome. Effect on skeletal development. J Bone Jt Surg ‐ Ser B. 1994;76(6):975‐981. 7983131

[10] Oussoren E , Brands MMMG , Ruijter GJG , van der Ploeg AT , Reuser AJJ . Bone, joint and tooth development in mucopolysaccharidoses: relevance to therapeutic options. Biochim Biophys Acta ‐ Mol Basis Dis. 2011;1812(11):1542‐1556. 10.1016/j.bbadis.2011.07.013 21827850

[11] Simonaro CM , D'Angelo M , He X , et al. Mechanism of glycosaminoglycan‐mediated bone and joint disease: implications for the mucopolysaccharidoses and other connective tissue diseases. Am J Pathol. 2008;172(1):112‐122. 18079441 10.2353/ajpath.2008.070564 PMC2189614

[12] Gentner B , Tucci F , Galimberti S , et al. Hematopoietic stem‐ and progenitor‐cell gene therapy for hurler syndrome. N Engl J Med. 2021;385:1929‐1940. 34788506 10.1056/NEJMoa2106596

[13] Parini R , Jones SA , Harmatz PR , Giugliani R , Mendelsohn NJ . The natural history of growth in patients with hunter syndrome: data from the hunter outcome survey (HOS). Mol Genet Metab. 2016;117:438‐446. 26846156 10.1016/j.ymgme.2016.01.009

[14] Grigull L , Beilken A , Schrappe M , et al. Transplantation of allogeneic CD34‐selected stem cells after fludarabine‐based conditioning regimen for children with mucopolysaccharidosis 1H (M. hurler). Bone Marrow Transplant. 2005;35(3):265‐269. 15580280 10.1038/sj.bmt.1704786

[15] Sauer M , Meissner B , Fuchs D , et al. Allogeneic blood SCT for children with Hurler's syndrome: results from the German multicenter approach MPS‐HCT 2005. Bone Marrow Transplant. 2009;43(5):375‐381. 18850023 10.1038/bmt.2008.328

[16] Weiner J , Lourie J . Practical Human Biology. Academic Press; 1981.

[17] Živičnjak M , Narančić NS , Szirovicza L , Franke D , Hrenović J , Bišof V . Gender‐specific growth patterns for stature, sitting height and limbs length in Croatian children and youth (3 to 18 years of age). Coll Antropol. 2003;27:321‐334. 12974162

[18] Robert‐Koch‐Institut . Referenzperzentile für anthropometrische Maßzahlen und Blutdruck aus der Studie zur Gesundheit von Kindern und Jugendlichen in Deutschland (KiGGS). 2nd ed. Beiträge zur Gesundheitsberichterstattung; 2013.

[19] Różdżyńska‐Świątkowska A , Jurecka A , Cieślik J , Tylki‐Szymańska A . Growth patterns in children with mucopolysaccharidosis I and II. World J Pediatr. 2015;11:226‐231. 25410665 10.1007/s12519-014-0517-6

[20] Bishop JR , Schuksz M , Esko JD . Heparan sulphate proteoglycans fine‐tune mammalian physiology. Nature. 2007;446:1030‐1037. 17460664 10.1038/nature05817

[21] Viskochil D , Clarke LA , Bay L , Keenan H , Muenzer J , Guffon N . Growth patterns for untreated individuals with MPS I: report from the international MPS I registry. Am J Med Genet Part A. 2019;179(12):2425‐2432. 31639289 10.1002/ajmg.a.61378 PMC6899772

[22] Pavone P , Praticò AD , Rizzo R , et al. A clinical review on megalencephaly: a large brain as a possible sign of cerebral impairment. Medicine. 2017;96(26):e6814. 28658095 10.1097/MD.0000000000006814 PMC5500017

[23] Kiely BT , Kohler JL , Coletti HY , Poe MD , Escolar ML . Early disease progression of hurler syndrome. Orphanet J Rare Dis. 2017;12(1):32. 28193245 10.1186/s13023-017-0583-7 PMC5307824

[24] Vellodi A , Young EP , Cooper A , et al. Bone marrow transplantation for mucopolysaccharidosis type I: experience of two British centres. Arch Dis Child. 1997;76(2):92‐99. 9068295 10.1136/adc.76.2.92 PMC1717089

[25] Weisstein JS , Delgado E , Steinbach LS , Hart K , Packman S . Musculoskeletal manifestations of hurler syndrome. J Pediatr Orthop. 2004;24(1):97‐101. 14676543 10.1097/00004694-200401000-00019

[26] Church H , Tylee K , Cooper A , et al. Biochemical monitoring after haemopoietic stem cell transplant for hurler syndrome (MPSIH): implications for functional outcome after transplant in metabolic disease. Bone Marrow Transplant. 2007;39(4):207‐210. 17220904 10.1038/sj.bmt.1705569

[27] Polgreen LE , Tolar J , Plog M , et al. Growth and endocrine function in patients with hurler syndrome after hematopoietic stem cell transplantation. Bone Marrow Transplant. 2008;41:1005‐1011. 18278070 10.1038/bmt.2008.20

[28] Polgreen LE , Thomas W , Orchard PJ , Whitley CB , Miller BS . Effect of recombinant human growth hormone on changes in height, bone mineral density, and body composition over 1‐2years in children with hurler or hunter syndrome. Mol Genet Metab. 2014;111(2):101‐106. 24368158 10.1016/j.ymgme.2013.11.013 PMC4018305

[29] Cattoni A , Motta S , Masera N , Gasperini S , Rovelli A , Parini R . The use of recombinant human growth hormone in patients with Mucopolysaccharidoses and growth hormone deficiency: a case series. Ital J Pediatr. 2019;45:93. 31370860 10.1186/s13052-019-0691-1 PMC6676577

[30] Ranke MB , Schwarze CP , Dopfer R , et al. Late effects after stem cell transplantation (SCT) in children – growth and hormones. Bone Marrow Transplant. 2005;35(1):77‐81. 15812537 10.1038/sj.bmt.1704853

[31] Bakker B , Oostdijk W , Bresters D , Walenkamp MJE , Vossen JM , Wit JM . Disturbances of growth and endocrine function after busulphan‐based conditioning for haematopoietic stem cell transplantation during infancy and childhood. Bone Marrow Transplant. 2004;33(10):1049‐1056. 15048143 10.1038/sj.bmt.1704481

[32] Guffon N , Pettazzoni M , Pangaud N , et al. Long term disease burden post‐transplantation: three decades of observations in 25 Hurler patients successfully treated with hematopoietic stem cell transplantation (HSCT). Orphanet J Rare Dis. 2021;16:1‐20. 33517895 10.1186/s13023-020-01644-w PMC7847591

[33] Szopa A , Domagalska‐Szopa M . Correlation between respiratory function and spine and thorax deformity in children with mild scoliosis. Med (United States). 2017;96:1‐5. 10.1097/MD.0000000000007032 PMC5459722 28562557

[34] Kosch M , Schaefer RM . Störungen des Säuren‐Basen‐Haushalts. Diagnostik und klinisches Bild. Deutsches Ärzteblatt. 2005;102:1896‐1899.

[35] Taylor DB , Blaser SI , Burrows PE , Stringer DA , Clarke JT , Thorner P . Arteriopathy and coarctation of the abdominal aorta in children with mucopolysaccharidosis: imaging findings. Am J Roentgenol. 1991;157:819‐823. 1909834 10.2214/ajr.157.4.1909834

