# Supplementary material for: Predictors of growth patterns in children with mucopolysaccharidosis I after haematopoietic stem cell transplantation
Source: JIMD Rep. 2022 Apr 26;63(4):371–8. doi: 10.1002/jmd2.12291 (PMC9259397; doi:10.1002/jmd2.12291)
Supplement: Supplementary file 1 — Data S1 Supporting information [file JMD2-63-371-s001.docx]

***Supplementary Table 1***

| **Patient** | **Sex** | **Origin** | **Age at transplantation (years)** | **IDUA^1^ mutation** | **Follow-up (years)** | **Enzyme activity at diagnosis (mU/ mg)^2^** |
| --- | --- | --- | --- | --- | --- | --- |
| 1 | f | Caucasian | 1.46 | Compound heterozygote c.1889C>T/ c.589+1G>A | 5.87 | 0.0071 |
| 2 | f | Caucasian | 1.64 | n.d. | 5.55 | 0.00 |
| 3 | f | Caucasian | 1.09 | homozygote c.208C>T (Q70X) | 6.94 | n.d. |
| 4 | f | Caucasian | 2.7 | homozygote p.W402X | 5.64 | 0.00 |
| 5 | f | Caucasian | 0.82 | compound heterozygote p.W402X/p.A79V | 15.94 | 0.00 |
| 6 | f | Caucasian | 2.28 | compound heterozygote p.W402X/p.A79V | 16.05 | 0.001 |
| 7 | m | Caucasian | 1.67 | compound heterozygote c.993delC/c.1154del21 | 13.88 | 0,00 |
| 8 | m | Caucasian | 0.81 | homozygote c.208>T (p.Q70X) | 11.79 | 0.00 |
| 9 | m | Caucasian | 2.18 | homozygote c.1205G>A/ p.(Trp402) | 1.36 | 0.002^3^ |
| 10 | f | Caucasian | 1.39 | compound heterozygote c.979G>C/ c.1099G>C | 11.37 | 0.00206 |
| 11 | m | Caucasian | 1.57 | n.d. | 9.2 | 0.00493 |
| 12 | f | Caucasian | 3 | homozygote p.Q70X | 8.62 | 0.00 |
| 13 | m | Caucasian | 3.08 | compound heterozygote W402X/ IVS1+G>A | 0.1 | 0.00^3^ |
| 14 | m | Caucasian | 2.15 | n.d. | 1.11 | 0.003 |

Characteristics of included MPS patients concerning ethnic, genetic and enzymatic information.
f, female; m, male; n.d., not done; ^1^ alpha-L-iduronidase; ^2^measured in leucocytes (reference range 0.15-0.38 mU/mg), ^3^data are given in nmol/h/mg protein

**Supplementary Table 2**

| **Patient** | **Donor** | **HLA match** | **Transplant** | **GvHD prevention** | **T-cell depletion** | **CD34+ x 10^6^/kg** | **CD3+ x 10^6^/kg** | **MNC x 10^8^/kg** |
| --- | --- | --- | --- | --- | --- | --- | --- | --- |
| 1 | MUD | 10/ 10 | PBSC | ATG 30mg/kg BW, MMF 30mg/kg BW | CD34-selection | 10 | 0.13 | 0.1202 |
| 2 | MUD | 10/ 10 | PBSC | ATG 30mg/kg BW | CD34-selection | 25.33 | 0.022 | 0.29 |
| 3 | MUD | 9/ 10 | PBSC | ATG 30mg/kg BW | CD34-selection | 31.05 | 0.0148 | 0.31 |
| 4 | Haplo (mother) | 5/ 8 | PBSC | ATG 40mg/kg BW, CSA 11mg/kg BW | CD34-selection | 54 | 0.0467 | - |
| 5 | MUD | 9/ 10 | PBSC | ATG 40mg/kg BW, CSA 11mg/kg BW | CD34-selection | 25 | 0.019 | - |
| 6 | MUD | 10/ 10 | PBSC | ATG 40mg/kg BW, CSA 11mg/kg BW | CD34-selection | 32 | 0.013 | 0.336 |
| 7 | Haplo  (mother) | 8/ 8 | PBSC | ATG 40mg/kg BW, CSA 11mg/kg BW | CD34-selection | 19.5 | 0.059 | - |
| 8 | MSD | 10/ 10 | BM | ATG 30mg/ kg BW, MTX 40mg/ m^2^,  CSA 3mg/kg BW | - | - | - | 3.33 |
| 9 | MUD | 10/ 10 | PBSC | ATG 30mg/ kg BW, MMF 45mg/ kg BW | α/β-depletion | 45.08 | 16.8025 | 10.724 |
| 10 | MUD | 9/ 10 | PBSC | ATG 30mg/ kg BW | CD34-selection | 16.4 | 0.017 | 0.17 |
| 11 | MUD | 10/ 10 | PBSC | ATG 30mg/ kg BW | CD34-selection | 13.78 | 0.0112 | 0.14 |
| 12 | MSD | 10/ 10 | BM | ATG 40 mg/ kg BW, CSA 5 mg/kg BW, MTX 10 mg/ m^2^ | - | 11.5 | 0.084 | 5.6 |
| 13 | MUD | 9/ 10 | BM | MTX 10mg/m^2^, CSA 3mg/ kg BW | - | 3.8 | 5.5 | 7.4 |
| 14 | MUD | 10/ 10 | PBSC | ATG 30 mg/kg BW, MMF 45 mg/kg BW | α/β-depletion | 20.55 | 8.9051 | 4.894 |

Detailed information about HSCT preparative regimen, donor and stem cell source.
MNC, mononuclear cells; MUD, matched unrelated donor; MSD, matched sibling donor; PBSC, peripheral blood stem cell; BM, bone marrow; ATG, antithymoglobulin; MMF, mycophenolate mofetil; CSA, ciclosporin; MTX, methotrexate; BW, Body Weight

**Supplementary Table 3**

| **Patient** | **Age at last follow-up (years)** | **Dysostosis multiplex^1^** | **Gibbus/ kyphosis^2^** | **Hip deformities^2^** | **Knee deformities^2^** | **Spinal cord compression^2^** | **Orthopaedic surgery (age at surgery)** | **Age at surgery for CTS (years)^3^** |
| --- | --- | --- | --- | --- | --- | --- | --- | --- |
| 1 | 7.33 | vertebra dysostosis multiplex | thoracic scoliosis | hip dysplasia, Coxa valga (CCD-angle 160° right, 170° left | Genua valga | no | Eight Plate epiphysiodesis (15.73) | no |
| 2 | 7.19 | hip dysostosis multiplex | Gibbus LV1 (kyphosis angle 26°); kyphosis angle TV1-LV3 66°, lumbar scoliosis (Cobb angle 18° LV1) | Coxa valga | Genua valga | no | no | 7.39 |
| 3 | 8.03 | vertebra dysostosis multiplex | Lumbar gibbus, thoracic-lumbar kyphoscoliosis (60°) | no | Genua valga | cranio-cervical junction (8mm) | no |  |
| 4 | 8.34 | vertebra and thoracic dysostosis multiplex | thoracic-lumbar kyphosis (Cobb angle 90°); scoliosis (Cobb angle 57°) | Coxa valga (CCD angle 153° right, 157° left) | no | no | no | 8.83 |
| 5 | 16.76 | no | throracic-lumbar kyphoscoliosis, Gibbus LV1/2 | no | no | stenosis foramen magnum (8mm sagittal) | no | no |
| 6 | 18.33 | thoracic vertebra dysostosis multiplex | thoracic-lumbar kyphoscoliosis (Cobb angle 20°), Gibbus | no | Genua valga | dysplasia cranio-cervical junction; foramen magnum 23 mm | no | 6.17 |
| 7 | 15.55 | no | kyphosis and Gibbus TV12/LV1 | hip dysplasia and luxation, Coxa valga | no | cranio-cervical junction (5mm) | Spondylodesis (10.57) | 8.3 |
| 8 | 12.6 | vertebra dysostosis multiplex | lumbar gibbus (Cobb angle 43°), throracolumbar scoliosis | hip dysplasia | Genua valga | moderate compression cranio-cervical junction | Eight Plate epiphysiodesis (9.82) | 3.59 |
| 9 | 3.54 | no | no | no | no | no | no | no |
| 10 | 12.76 | no | thoracic kyphosis TV1 (Cobb angle 30°) and Gibbus | hip dysplasia; AC angle right 27° and 30° left) | Genua valga | moderate compression cranio-cervical junction | Corrective osteotomy knee (7.64) | 7.64 |
| 11 | 10.77 | dysostosis multiplex (vertebra, hands, pelvis) | lumbar kyphoscoliosis (Cobb angle 48°) and gibbus, thoracic scoliosis (Cobb angle 11°) | hip dysplasia and luxation, AC angle 40° (right) | Genua valga | moderate compression cranio-cervical junction | no | 9.22 |
| 12 | 11.62 | thoracic dysostosis multiplex | gibbus LV2 (Cobb angle 34°); thoacic-lumbar scoliosis (Cobb angle 51°) | hip dysplasia and luxation | no | moderate compression cranio-cervical junction | no | 9 |
| 13 | 3.18 | no | no | no | no | no | no | no |
| 14 | 3.25 | no | no | hip dysplasia, coxa valga | no | moderate compression cranio-cervical junction | no | no |

Description of the orthopaedic pathologies and surgery of this MPS cohort. ^1^ radiologically determined; ^2^ radiologically determined by different observers, data not available for all patients; CTS, carpal tunnel syndrome; ^3^ pathology in MPS patients: bone dysplasia, GAG deposition inside the carpal tunnel and thickened flexor retinaculum

**Supplementary Table 4**

| **Patient** | **WBC > 1000/μl day** | **ANC > 500/μl day** | **Platelets > 50000/nl day** | **aGvHD grade^1^** | **cGvHD grade** | **Last chimerism analysis (%)** | **Worst chimerism analysis (%)** | **Additional cell therapy performed (DLI)** |
| --- | --- | --- | --- | --- | --- | --- | --- | --- |
| 1 | 14 | 17 | 23 | 1 (skin) | 0 | 100 | 100 | no |
| 2 | 13 | 17 | 11 | 0 | 0 | 100 | 99 | no |
| 3 | 13 | 11 | 15 | 0 | 0 | 99 | 95 | no |
| 4 | 9 | 10 | 12 | 0 | 0 | 99 | 84 | no |
| 5 | 18 | 21 | * | 0 | 0 | 98 | 62 | 3 x 10^4^ CD3+/ kg  (d + 84), (d + 196) |
| 6 | 11 | 16 | 9 | 0 | 0 | 92 | 51 | 3,08 x 10^4^ CD3+/ kg (d + 77) |
| 7 | 12 | 14 | 20 | 0 | 0 | 97 | 88 | no |
| 8 | 26 | 28 | 16 | 0 | 0 | 85 | 81 | 3 x 10^4^ CD3+/ kg  (d + 335) |
| 9 | 10 | 12 | 9 | 0 | 0 | 100 | 100 | no |
| 10 | 15 | 20 | 14 | 1 (skin) | 2^2^ (intestinal) | 98 | 45 | 5 x 10^5^ CD3+/ kg (d + 38) |
| 11 | 17 | 21 | 14 | 0 | 0 | 99 | 99 | no |
| 12 | 16 | 16 | 26 | 1 (skin) | 0 | 97 | 95 | no |
| 13 | 20 | 21 | 23 | 0 | 0 | 100 | 100 | DLI in another center |
| 14 | 16 | 20 | 11 | 0 | 0 | 99 | 98 | no |

*Engraftment and GvHD data of the MPS cohort with information about additional cell therapy.
ANC, absolute neutrophil count; aGvHD, acute graft-versus-host disease; cGvHD, chronic graft-versus-host disease; DLI, donor lymphocyte infusion; WBC, white blood cell count; ^1^ after HSCT, only topical steroid treatment; ^2^ after DLI,* *resolved after 6 months after mesenchymal stem cells; * at discharge 40.000/μl*

**Supplementary Table 5**

| **Patient** | **Age at diagnosis (years)** | **Last enzyme activity^1^ in nmol/h/mg protein** | **Worst enzyme activity^1^ in nmol/h/mg protein** | **MeanTSH  in mU/l  (ft4 in ng/dl)** | **Last GFR in ml/min** | **Hydrocephalus**  **(Age at shunt surgery)** | **Heart involvement** | **Age at menarche**  **(years)** |
| --- | --- | --- | --- | --- | --- | --- | --- | --- |
| 1 | 0.64 | 1.48 | 0.63 | n.d. | 196 | no | cardiomyopathy | Prepubertal* |
| 2 | 1.21 | 6.28 | 6.28 | 2.98 (1.23) | 134 | Internal and external Hydrocephalus (1.62) | no | Prepubertal* |
| 3 | n.d. | n.d. | n.d. | 3.68 (0.99) | 139 | No | no | Prepubertal* |
| 4 | 0.42 | 1.74 | 0.28 | 2.57 (1.53) | 180 | Hydrocephalus aresorptivus (5.28) | AI, MI | Prepubertal* |
| 5 | 0.19 | 0.72 | 0.56 | 2.71 (1.08) | 132 | Internal Hydrocephalus  (no surgery) | no | 14.03 |
| 6 | 1.34 | 1.78 | 1.34 | 1.56 (0.97) | 146 | Internal Hydrocephalus  (no surgery) | no | 16.32 |
| 7 | 1.23 | 0.49 | 0.47 | 2.75 (1.19) | 196 | Internal Hydrocephalus (1.57) | MI, TI |  |
| 8 | 0.46 | 2.99 | 0.39 | 2.65 (1.27) | 185 | Internal Hydrocephalus (0.86) | MI |  |
| 9 | 1.58 | n.d. | n.d. | 4.87 (1.1) | 190 | no | MI |  |
| 10 | 1.04 | n.d. | n.d. | 4.26 (1.03) | 184 | no | AI, MI | 12.85 |
| 11 | 1.64 | 4.86 | 4.86 | 2.73 (0.99) | 136 | no | MI, TI |  |
| 12 | 0.59 | 3.02 | 1.36 | 2.49 (1.14) | 178 | Internal Hydrocephalus  (no surgery) | AI, MI | Prepubertal* |
| 13 | n.d. | 0.48 | 0.48 | 3.1 (1.3) | 169 | no | No |  |
| 14 | 1.72 | n.d. | n.d. | 4.02 (1.27) | 217 | no | Cardiomyopathy |  |

Information about enzyme activity and kidney function of the patients, which in our study have no impact in growth parameters. ^1^ Alpha-L-iduronidase activity after HSCT, measured in leucocytes (reference range 0.27-9 nmol/h/mg protein, measured enzyme activity at follow up in dried blood spot and at diagnosis in heparin-blood (different measurement); n.d., not done; * at last follow-up

**Supplementary Table 6**

|  | | **Date of measurement** | **Weight SDS (range)** | **Length SDS (range)** | **Head circumference SDS (range)** |
| --- | --- | --- | --- | --- | --- |
| All patients | | Birth | 0.28 (-1.08 to 1.53) | 0.22  (-1.72 to 1.72) | 0.32  (-1.24 to 1.56) |
|  |  | 24 months | 0.10  (-1.45 to 1.94) | -1.23  (-3.51 to 1.00) | 1.82  (-1.45 to 1.94) |
| Age at transplantation | < 2 years | 24 months | 0.94  (-1.12 to 1.94) | 0.4  (-3.48 to 1.00) | 1.52  (-1.05 to 2.43) |
|  | > 2 years |  | -0.89  (-1.45 to 0.29) | -2.83  (-3.51 to 0.25) | 2.88  (1.31 to 4.65) |
| Type of donor | unrelated | 24 months | -0.11  (-1.21 to 1.48) | -1.13  (-3.51 to 0.74) | 1.52  (-1.05 to 4.65) |
|  | related |  | 1.23  (-1.45 to 1.94) | 0.47  (-2.86 to 1.00) | 2.94  (2.43 to 3.45) |

Mean Standard deviation scores in Infancy

**Supplementary Table 7**

|  | **Groups** | **Length SDS** | **p-value** | **Sitting height SDS** | **p-value** | **Leg length SDS** | **p-value** |
| --- | --- | --- | --- | --- | --- | --- | --- |
| Age at transplantation | Younger than 2 years | -2.25  (-2.76 to -1.69) | < 0.01 | -2.80  (-3.54 to -2.05) | 0.03 | -1.60  (-2.24 to -0.96) | 0.10 |
|  | Older than 2 years | -3.71  (-4.58 to -2.84) |  | -4.38  (-5.61 to -3.15) |  | -2.41  (-3.36 to -1.37) |  |
| Type of donor | Related | -2.66  (-3.74 to -1.58) | 0.70 | -3.28  (-4.61 to -1.96) | 0.80 | -2.17  (-3.15 to -1.18) | 0.30 |
|  | Unrelated | -2.50  (-3.18 to -1.82) |  | -3.15  (-4.13 to -2.17) |  | -1.66  (-2.37 to -0.95) |  |

|  | **Groups** | **Weight SDS** | ***p*-value** | **Head circumference SDS** | ***p*-value** |
| --- | --- | --- | --- | --- | --- |
| Age at transplantation | Younger than 2 years | -0.62  (-1.26 to 0.03) | 0.04 | 0.62 (-0.60 to 1.84) | 0.40 |
|  | Older than 2 years | -1.87  (-2.92 to 0.82) |  | 1.43 (-0.57 to 3.42) |  |
| Donor | Related | -1.21  (-2.36 to 0.61) | 0.30 | 2.49 (1.35 to 3.62) | < 0.01 |
|  | Unrelated | -0.57 (-1.3 to 0.17) |  | -0.09 (-0.95 to 0.76) |  |

*Estimated marginal means, non-adjusted.*

*Data are presented as estimated marginal means (95% confidence intervals); p values are based on the linearly independent pairwise comparisons among the estimated marginal means*

***Supplementary Figure 1*** *Distribution of mean SDS for weight, body length and head circumference the first two years in (A) the whole cohort, (B) cohort split by age at HSCT and (C) cohort split by donor type.*


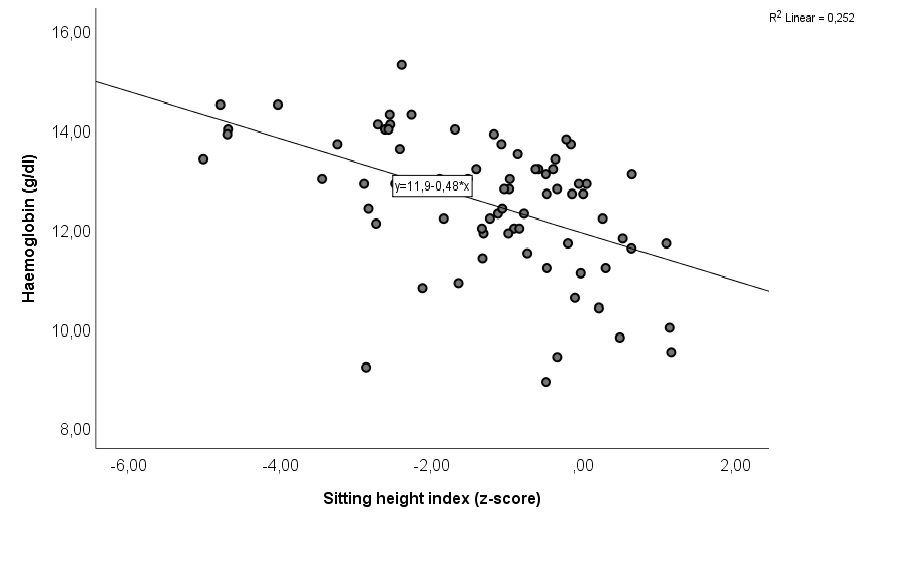
***Supplementary Figure 2*** *Haemoglobin as function of sitting height index (z-score)*
